# Redefining the Roles of Aspirin across the Spectrum of Cardiovascular Disease Prevention

**DOI:** 10.2174/1573403X19666230502163828

**Published:** 2023-10-02

**Authors:** Matthew T. Brown, Kristina S. Bortfeld, Laurence S. Sperling, Nanette K. Wenger

**Affiliations:** 1 Division of Cardiology, Department of Medicine, Emory University School of Medicine, Atlanta, Georgia;; 2 Department of Medicine, Emory University School of Medicine, Atlanta, Georgia

**Keywords:** Aspirin, atherosclerotic cardiovascular disease, coronary artery disease, mechanical heart valves, preeclampsia, primary prevention, secondary prevention

## Abstract

Even before its role in platelet inhibition was fully characterized in the 1980s, aspirin had been incorporated into the cardiovascular disease care algorithm. Early trials examining its use in unstable angina and acute myocardial infarction revealed evidence of its protective role in the secondary prevention of atherosclerotic cardiovascular disease (ASCVD). Large trials assessing use in the primary prevention setting and optimal dosing regimens were studied in the late 1990s and early 2000s. As a cornerstone of cardiovascular care, aspirin was incorporated into primary and secondary ASCVD prevention guidelines in the United States and mechanical heart valve guidelines. However, in recent years, with significant advances in medical and interventional ASCVD therapies, scrutiny has been placed on the bleeding profile of aspirin, and guidelines have adapted to new evidence. Updates in primary prevention guidelines reserve aspirin only for patients at higher ASCVD risk and low bleeding risk - though questions remain in ASCVD risk assessment as risk-enhancing factors have proven difficult to incorporate on a population level. New thoughts regarding aspirin use in secondary prevention - especially with the concomitant use of anticoagulants - have altered recommendations as additional data accrued. Finally, a recommendation for aspirin and vitamin K antagonists with mechanical heart valves has been modified. Despite aspirin losing a foothold in cardiovascular care, new evidence has strengthened claims for its use in women at high risk for preeclampsia.

## 1 INTRODUCTION

Aspirin has been the cornerstone of cardiovascular disease prevention for decades. However, recent evidence requires that its role be redefined across the spectrum of cardiovascular disease prevention. Recent recommendations for the primary prevention of atherosclerotic cardiovascular disease (ASCVD) have reduced the age for initiation of aspirin in high-risk individuals [1-3], and data for secondary prevention of coronary artery disease favor alternative anti-platelet agents long-term [4, 5]. In addition, the most recent mechanical heart valve guidelines report little quality evidence for using aspirin added to coumadin [6, 7]. A new cardiovascular indication for aspirin in cardio-obstetrics is the prevention of preeclampsia [8]. With this changing landscape, it is important to understand the historical basis for aspirin use in the cardiovascular arena, to trace its roots in early United States guidelines and to rationalize the application in contemporary practice.

## 2 PRIMARY PREVENTION OF ASCVD

Given the recent United States Preventive Services Task Force (USPSTF) recommendation on aspirin use for the primary prevention of ASCVD, it is important to define the diseases encompassing ASCVD and differentiate the indications for aspirin use, as primary and secondary prevention approaches are distinct. ASCVD refers to three broad categories of cardiovascular disease: (1) coronary heart disease, including myocardial infarction (MI), stable or unstable angina, and coronary stenosis, (2) cerebrovascular disease, including transient ischemic attack (TIA), ischemic stroke, and carotid artery stenosis, and (3) peripheral arterial disease (PAD) including aortic aneurysm, claudication, and revascularization of peripheral arteries. Primary prevention specifically pertains to preventing the first occurrence of any of the above conditions in unaffected, asymptomatic individuals by identifying risk factors and instituting interventions.

The USPSTF updated recommendation statement emphasizes shared decision-making for low-dose aspirin initiation in adults aged 40 to 59 years with ≥ 10% American College of Cardiology/American Heart Association (ACC/ AHA) Pooled Cohort Equation for estimation of 10-year ASCVD risk [3, 9]. Data are inconsistent for patients with risk-enhancing factors leaving unanswered questions in high-risk primary prevention populations such as patients with chronic inflammatory diseases, diabetes-associated comorbidities, and significant subclinical atherosclerosis as those with coronary artery calcium scores > 300 that may not be captured by the pooled cohort equation [10, 11]. The statement cites a small net benefit in qualifying persons not at risk for bleeding continuing aspirin until age 75 but concludes no net benefit of initiating aspirin in adults aged ≥ 60 years. With this reduction in the age of aspirin initiation benefit compared to its prior 2009 statement supporting aspirin for MI reduction in men between ages 45 to 79 and ischemic stroke reduction in women between ages 55 to 79, the USPSTF is aligned with the ACC/AHA and American Diabetes Association (ADA) guidelines. In 2019, ACC/AHA updated primary prevention guidelines and added the age of benefit from 40 to 70 years for aspirin initiation while maintaining original 2002 recommendations for use in elevated, ≥ 10% ASCVD 10-year risk individuals without increased risk of bleeding [1, 12]. The following year the ADA modified its recommended age of aspirin initiation benefit to any gender aged 50 to 70 years old from its original 2007 statement for use in patients with diabetes and elevated ASCVD risk > 40 years old, and the 2010 consensus update offering sex-specific criteria [2]. Internationally, the European Society of Cardiology (ESC) recommended against antiplatelet therapy initiation among low/moderate ASCVD risk individuals reserving consideration only for those with diabetes and high or very high ASCVD risk due to the increased risk of major bleeding [13]. Each organization emphasizes the use of low-dose aspirin, 75 - 162 mg daily, to minimize bleeding risk and remark on avoidance in patients with increased risk for bleeding, citing a history of gastrointestinal bleeding, peptic ulcer disease, bleeding at other sites, chronic kidney disease, coagulopathy/thrombocytopenia, and medication use such as anticoagulants, steroids, and nonsteroidal anti-inflammatory drugs as factors to consider.

### 2.1 Relevant Studies

Data have driven these guideline recommendations. The earliest statements were based on the Physician’s Health Study of 1989, the Women’s Health Study of 2005, and a few others [14-18]. Updated statements are based on a series of randomized controlled trials since 2018, namely, A Study of Cardiovascular Events in Diabetes (ASCEND), Aspirin in Reducing Events in the Elderly (ASPREE), and Aspirin to Reduce Risk of Initial Vascular Event (ARRIVE) [19-22]. Each of these trials recruited over ten thousand participants to investigate the role of aspirin in the primary prevention of cardiovascular disease.

The Physician’s Health Study of 1989 followed over 22,000 male participants for 5 years, comparing aspirin 325 mg every other day to placebo and revealed a 44% relative risk (RR) reduction in MI, without any differences in all-cause or cardiovascular death but with significant bleeding (RR 1.32) and transfusion need (RR 1.71) [14]. The Women’s Health Study of 2005 followed over 39,000 women ≥ 45 years old for 10 years, comparing aspirin 100 mg every other day to placebo and determined a 17% relative risk reduction in stroke (largely driven by ischemic etiology) without effects on fatal MI or cardiovascular disease death, although with significant increased gastrointestinal bleeding requiring transfusion (RR 1.4) [15]. The USPSTF 2009 statement with separate recommendations for men and women reflects these data, with the important consideration for evaluating bleeding risk when initiating aspirin.

The updated USPSTF statement incorporates revisions based on the series of 2018 trials and others. ASCEND enrolled over 15,000 patients with diabetes but no ASCVD to compare aspirin 100 mg daily to a placebo for over 7 years of follow-up [20]. Efficacy endpoints were achieved in the aspirin group with a significant reduction in serious vascular events defined as MI, TIA/stroke, or cardiovascular death with 658 (8.5%) *vs.* 743 (9.6%), RR 0.88 [0.79 - 0.^97^]. Safety endpoints were also significant for increased rates of major bleeding events - most often gastrointestinal followed by other extracranial sources - with 314 (4.1%) *vs.* 245 (3.2%) RR 1.29 (1.09 - 1.52). ASPREE enrolled over 19,000 participants over 65 without dementia or ASCVD to compare the effects of aspirin 100 mg daily to placebo for nearly 5 years [21, 22]. The primary outcome was no difference in disability free-survival 10.7 v 11.3 events, hazard ratio (HR) 0.95 [0.83 - 1.0^8^] with concerning secondary outcomes of higher major hemorrhage 8.6 *vs.* 6.2 events, HR 1.38 (1.18 - 1.62) and higher all-cause mortality 12.7 *vs.* 11.1 events, HR 1.14 (1.01 - 1.29) largely driven by higher cancer rates among the aspirin cohort (3.1% v 2.3%). Recent work analyzed a subset of the 12,815 ASPREE participants that underwent lipoprotein(a) genotyping to identify 406 (3.2%) elderly patients with the rs3789220-C LPA gene variant and 2,556 (19.9%) patients with high 43-variant lipoprotein (a) genetic risk scores (LPA-GRS) each previously shown to correlate with elevated plasma lipoprotein(a) levels known to confer up to 4-fold increased risk of cardiovascular disease [23-31]. A comparison of aspirin use to placebo among these genetically predisposed, higher-risk elderly patients to non-carriers or low LPA-GRS scores revealed a significant reduction in major adverse cardiovascular events (MACE) among rs3798220-C carriers [3.6 v 15 events per 1,000 person-years; HR 0.24 (0.07 - 0.82); p = 0.024), a non-statistically significant risk attenuation among high LPA-GRS patients [7.5 *vs.* 10.8 events per 1,000 person-years; HR 0.73 (0.48-1.10); p = 0.128], and no difference in clinically significant bleeding events among either small group of genetically predisposed higher risk elderly patients [31]. ARRIVE recruited over 12,000 patients with estimated moderate ASCVD risk (mean 10-year risk 17%), although overall event rates were much lower than anticipated, with 4.29% occurring among the aspirin group and 4.48% among the placebo group without significant differences among MI (1.4% *vs.* 1.6%) or stroke (1.2% *vs.* 1.1%) [19]. Among this lower-risk-than-anticipated population, gastrointestinal bleeding was significantly increased among aspirin users (0.97% *vs.* 0.46%, p ≤ 0.001), although the risk of total (HR 0.53) and nonfatal MI (HR 0.55) was reduced with a particularly high relative risk reduction of 82% among the 50- to 59-year-old subset.

The USPSTF pooled trial data of these and others to understand the potential benefits and harms of low-dose aspirin in the primary prevention setting [3, 15, 17-20, 22, 25-28, 32]. Their consolidated review revealed a statistically significant decreased risk of nonfatal MI (11 trials, n = 134 470, OR 0.88 [0.80 - 0.^96^]) and ischemic stroke (5 trials, n = 54 947, OR 0.88 [0.78 - 1.00, p = 0.0^46^]) although a significantly increased risk of major gastrointestinal bleeding (10 trials, n = 119 130, OR 1.58 [1.38 - 1.^80^]) and intracranial bleeds (11 trials, n = 134 470, OR 1.31 [1.11 - 1.^54^]). With low fatality rates overall, no significant difference was found among aspirin use in rates of fatal MI, ischemic or hemorrhagic stroke, major gastrointestinal bleeding, and cardiovascular or all-cause mortality. When bleeding occurred, it happened relatively quickly after aspirin initiation, and the absolute incidence of bleeding increased with age, particularly in adults 60 years or older. Microsimulation modeling found modest net positive life-years and quality-adjusted life-years for men and women with ≥ 10% 10-year ASCVD risk among 40 to 59 years old initiating aspirin for primary prevention and little incremental benefit in continuing beyond 75 to 80 years [24, 33]. With generally negative life-years gained across all 10-year ASCVD risk levels in men and women ≥ 60 years old initiating aspirin, the USPSTF finalized its recommendations with moderate certainty evidence (Table **1**).

## 3 SECONDARY PREVENTION OF ASCVD

Once called “the wonder drug that nobody understands,” shortly after its synthesis and commercialization in 1897, aspirin’s role in platelet inhibition eventually earned a trio of scientists the 1982 Nobel Prize in the Physiology of Medicine [34]. The following year the Veteran’s Affairs (VA) Cooperative Study investigated aspirin’s role in 1266 men with unstable angina across 12 VA Hospitals, with 625 randomized to receive aspirin 324 mg and 641 to receive a placebo [35]. The frequency distribution of death or acute myocardial infarction during the 12-week study period was striking, with a 51% reduction in the aspirin group (31 [5.0%] *vs.* 65 [10.1%], p 0.0002). In 1988, the Second International Study of Infarct Survival (ISIS-2) reported an absolute mortality reduction of 2.4 per 100 for a number needed to treat 42 among the aspirin monotherapy arm of a 2 x 2 factorial study evaluating a one-time 1.5 million unit IV streptokinase dose and/or 30 days of aspirin 160 mg within 24 hours of chest pain onset among a subset of 17,187 patients with suspected acute MI across 417 European hospitals [36]. A variety of trials were consolidated in a 1994 publication by the Antiplatelet Trialists’ Collaboration reviewing 145 randomized-controlled trials comparing antiplatelet agents to placebo and 29 comparing different antiplatelet agents [37]. Though now outdated, resultant data on antiplatelet (AP) use among over 70,000 “high risk” patients with either acute MI (n ≈ 20,000), prior MI (n ≈ 18,000), prior stroke or TIA (n ≈ 11,000), and other “high risk” conditions (n ≈ 22,000) including unstable angina (n ≈ 4,000), stable angina, a chronic coronary disease with or without coronary bypass grafting or percutaneous intervention, atrial fibrillation, valvular heart disease, a peripheral vascular disease with or without angioplasty/vascular surgery, diabetes, and hemodialysis not only helped define the conditions that constitute the spectrum of ASCVD today but also established the fundamentals of secondary prevention antiplatelet use for the 21st century. Among antiplatelet agents studied, the most widely tested was “medium dose” aspirin, 75 - 325 mg daily, which worked as effectively as higher aspirin doses as well as six other AP regimens. The trialists remarked on using an aspirin loading dose (160 - 325 mg) out of an abundance of caution in emergencies. They commented on insufficient long-term data, although protective effects were maintained after 3 years of therapy in some trials. Reviewing all 70,000 high-risk patients, AP use significantly reduced rates of non-fatal MI and non-fatal stroke by approximately 1/3rd and vascular death by approximately 1/6th. Likewise, vascular events were significantly reduced by nearly 1/4th in each sub-group of the high-risk cohorts (p ≤ 0.00001) - acute MI (10% AP *vs.* 14% Placebo), prior MI (13% AP *vs.* 17% Placebo), prior stroke/TIA (17% AP *vs.* 22% Placebo), and unstable angina (9% AP *vs.* 14% Placebo) plus others (6% AP *vs.* 8% Placebo) - and, remained significant when further stratifying by middle/old age, hyper/normotensive, and diabetic/non-diabetic. Notably, overall mortality - death from any cause - was also significantly reduced among the high-risk subgroups using AP agents. A 2002 Antiplatelet Trialists’ Collaboration review updated the dataset to include a total of 287 randomized-controlled trials with over 135,000 patients in AP *vs.* placebo studies and 77,000 undergoing AP comparisons [38]. The dataset largely confirmed aspirin’s protective effects against future vascular events. It supported the effectiveness of lower doses (75 - 150 mg daily) compared to higher doses and long-term benefits in patients experiencing MI, stroke, or TIA. With a larger dataset comparing AP regimens, including combinations of aspirin and other APs to aspirin monotherapy, it became apparent that clopidogrel could be used as an effective alternative and that short-term intravenous use of glycoprotein IIa/IIIb antagonists in combination with aspirin during percutaneous intervention offered benefit but at an increased risk of bleeding. The review also tabulated the risk of major extracranial bleeds with an increased total odds ratio of 1.6 among AP use in different high-risk populations.

Since the 2001 AHA/ACC Guidelines for Preventing Heart Attack and Death in Patients with ASCVD Update, aspirin (75 - 325 mg daily) has appeared in secondary prevention guidelines with a 2006 update introducing dual-antiplatelet therapy (DAPT) for 12 months after coronary stenting that has been maintained in the recent 2011 update which also incorporates aspirin’s role in preventing saphenous vein graft closure after coronary artery bypass grafting (CABG) and managing symptomatic lower-extremity PAD [39-46]. The 2011 update supported an aspirin 75 - 162 mg recommendation for all patients with coronary artery disease (IA) - unless intolerant/allergic, in which case substituting clopidogrel 75 mg (IB) - even in the presence of an indication for anticoagulation (IA), noting the increased risk of bleeding requiring close monitoring [41, 47-51]. There is also a class IIb recommendation for DAPT with daily aspirin 75-162 mg and clopidogrel 75 mg for stable coronary artery disease [52]. The 2016 ACC/AHA Guideline Focused Update on Duration of DAPT in Patients with coronary artery disease recommended an indefinite aspirin dose of 81 mg in combination with varying durations of P2Y12 inhibitors depending on the situation (acute coronary syndrome *vs.* stable ischemic heart disease), treatment (medical, lytic, PCI, and/or CABG), and bleeding risk with a brief reference to limited duration triple therapy as necessary [53]. The class IIb consideration for continued DAPT after 12 months in the absence of bleeding issues was maintained, but an added caveat addressing increased bleeding risk in the setting of concomitant treatment with oral anticoagulation factors into the decision to discontinue P2Y12 therapy at earlier time points. With the introduction of newer-generation drug-eluting stents (DES) associated with lower rates of stent thrombosis and in-stent restenosis, there has been much scrutiny into the nuances of antiplatelet therapy - especially in regard to DAPT duration and preferred long-term maintenance agents/dosing with and without indications for oral anticoagulants [54, 55].

Atrial fibrillation is a frequent comorbidity among patients with CAD and warrants consideration of bleeding risk when selecting appropriate secondary prevention therapy. With regard to concomitant antiplatelet therapy with oral anticoagulation (OAC) indications, the most up-to-date recommendations initially came from the 2018 Joint European Consensus Document on the Management of Antithrombotic Therapy in Atrial Fibrillation Patients presenting with ACS and/or undergoing PCI citing several key randomized trials [56-60]. This European Consensus provides three pathways for management within the first 12 months post-PCI depending on the relative thrombotic and bleeding risks: [Bullet Formatting Start] - High Thrombotic: OAC + DAPT x 1 - 6 month(s) before OAC/Clopidogrel - High Bleeding: OAC + DAPT x 1 month then OAC/Clopidogrel - Excessive Bleeding: OAC/Clopidogrel only [Bullet Formatting End]

Regardless of the regimen during the initial 12 months, the document suggests one final common pathway for long-term management using only chronic maintenance anticoagulation without either aspirin or P2Y12 inhibitor use [61, 62]. A North American Perspective was soon to follow in 2018, similarly outlining three possible pathways focused on limited duration triple therapy (OAC + DAPT) for varying degrees of ischemic/thrombotic and/or bleeding risks with a transition to OAC monotherapy after 12 months [63]. While the 2018 North American Perspective did not commit to a particular AP agent of choice like its European counterpart, a 2021 update clarifies the preferred AP agent for use with OAC in the first 12 months following PCI as a P2Y12 inhibitor rather than aspirin, recommending clopidogrel for routine use and reserving ticagrelor for patients at high thrombotic and acceptable bleeding risks [64]. These recommendations propose shorter-duration AP/OAC combination therapy algorithms to minimize bleeding risk while effectively preventing ischemic/thrombotic events. It is also important to understand this balance in those without indications for OAC. Two recent systematic reviews and meta-analyses have focused on determining the optimal duration of DAPT to achieve this balance in patients with CAD undergoing PCI without indications for OAC [65, 66]. Each includes the same five RCTs comparing standard 12 months of DAPT to 1-3 month shorter DAPT with P2Y12 continuation though the initial 2020 review analyzes all 32,145 patients presenting for PCI with either stable coronary disease or acute coronary syndrome while the 2021 review focuses solely on the 18,046 patients presenting urgently with acute coronary syndrome [67-71]. Neither analysis found a significant difference in MACE using the shorter DAPT duration and P2Y12 monotherapy continuation, while both reported significant reductions in major bleeding by about 40% compared to the traditional 12 months of DAPT. The acute coronary syndrome-focused study also reported an all-cause mortality benefit (1.0% *vs.* 1.42%, OR 0.71 [0.53 - 0. 95], p < 0.001) that was not replicated in the larger, mixed cohort of stable and acute coronary disease. With this evidence to suggest less bleeding without an increase in adverse ischemic/thrombotic outcomes following PCI, the latest European and American revascularization guidelines have incorporated consideration for shorter-duration DAPT of 3-6 months and 1-3 months, respectively, followed by P2Y12 inhibitor monotherapy as 2a recommendations [72, 73]. The class 1a recommendation remains for 12 months of DAPT after PCI in those that can tolerate it, but with this new evidence, the typical transition to long-term aspirin monotherapy for secondary prevention is currently in question. First, for patients years after any ASCVD event currently maintained on aspirin, a large-scale study was published in 2021 by the ADAPTABLE study team of a unique open-label, randomized control trial recruiting over 15,000 patients via PCOR.net to assess the comparative effectiveness of aspirin 81 mg *vs.* 325 mg in patients with established ASCVD [74]. No significant difference was observed in rates of death or hospitalization for MI, stroke, or major bleeding between the two groups, although 41% of the aspirin 325 mg group switched their assigned dose during the 42-month study. In patients at the 12-month mark of DAPT ready to transition to a single agent, another randomized controlled trial, HOST-EXAM, compared continuation of clopidogrel 75 mg *vs.* aspirin 100 mg monotherapy after tolerating 6 - 18 months of DAPT following PCI in 5,438 patients across 37 sites in South Korea [5]. The majority of patients in each treatment group underwent PCI for unstable angina (≈ 35%), NSTEMI (≈ 19%), or STEMI (≈ 17%) and had similar baseline comorbidities with comparable coronary complexity necessitating PCI for bifurcation lesions in 10% of cases and for CTO in 9%. The 2,710 patients treated with clopidogrel monotherapy had significant reductions in readmission due to ACS [66 (2.5%) *vs.* 109 (4.1%); HR 0.63 (0.45 - 0.82); p = 0.00^1^], stroke [18 (0.7%) *vs.* 43 (1.6%); HR 0.42 (0.24 - 0.73); p = 0.00^2^] driven by hemorrhagic subtype [4 (0.2%) *vs.* 17 (0.6%)], and any bleeding [61 (2.3%) *vs.* 87 (3.3%); HR 0.70 (0.51 - 0.98); p = 0.0^36^] driven by major bleeding [33 (1.2%) *vs.* 53 (2.0%] compared to the 2,728 patients treated with aspirin monotherapy. No significant differences among all-cause death, cardiac death, non-cardiac death, non-fatal MI, need for repeat revascularization, or in-stent thrombosis rates between monotherapy groups. This study has been incorporated into two recent meta-analyses along with several other large randomized controlled trials to better evaluate any benefits and/or risks of long-term P2Y12 inhibitor use for secondary prevention of ASCVD. The larger meta-analysis reviews 9 RCTs encompassing a total of 61,623 patients with various qualifying types of ASCVD (39.5% stroke, 29.9% acute coronary syndrome, 20.1% chronic coronary syndrome, and 10.5% peripheral artery disease) and finds significant reductions in MACE (RR 0.89 [0.84 - 0.^95^], p = 0.0003) and MI (RR 0.81 [0.71 - 0.^92^], p = 0.0009) without any differences in rates of stroke, bleeding, or all-cause mortality among 30,844 patients continued on a P2Y12 inhibitor compared to the 30, 779 patients randomized to aspirin [5, 50, 67, 75-81]. Specifically related to patients with coronary disease, the recent PANTHER (P2Y12 inhibitor versus Aspirin moNoTHERapy in patients with coronary artery disease) meta-anlaysis presented at the 2022 European Society of Cardiology meeting analyzed 7 of the same RCTs to compare outcomes among 24,325 patients with established CAD without a need for OAC randomized to either long-term P2Y12 inhibitor (62% clopidogrel, 38% ticagrelor) or aspirin monotherapy for median 557 days [5, 50, 75-77, 80-82]. Similar to the larger meta-analysis, the PANTHER study reported a significant primary efficacy outcome for the reduction in MACE for P2Y12 inhibitors (5.5% *vs.* 6.3%, HR 0.88 [0.79 - 0.^97^], p = 0.014) while no difference in major bleeding, stroke, cardiovascular death, or all-cause mortality was found. The driving factor for MACE reduction could be attributed to a significant reduction in MI (HR 0.77, [0.66 - 0.^90^], p < 0.001) using long-term P2Y12 inhibitor as opposed to aspirin with notable secondary outcomes revealing significant reductions in both definite (0.42 [0.19 - 0.^97^], p = 0.41) and definite/probable stent-thrombosis (0.46 [0.23 - 0.^92^], p = 0.028). Subgroup analysis for primary efficacy did not reveal significant differences between clinical presentation, bleeding risk based on Precise DAPT score, body mass index, geographic region, proton-pump inhibitor use, or type of P2Y12 inhibitor, though it was pertinent for a non-significant trend toward benefit among the type of revascularization with P2Y12 inhibitor monotherapy for PCI found to have an HR 0.70 (0.56 - 0.86). Although major bleeding was not different, rates of hemorrhagic stroke (0.32 [0.14 - 0.^75^], p = 0.009) and gastrointestinal bleeding (0.75 [0.57 - 0.^97^], p = 0.027) were significantly reduced among P2Y12 inhibitor monotherapy also contributing to a significant reduction in the net adverse clinical events (NACE) key secondary outcome (6.4% *vs.* 7.2%, HR 0.89 [0.81 - 0.^98^], p = 0.02). Evidence continues to accrue in favor of P2Y12 inhibitors for shorter-duration DAPT, use with OACs, and now as long-term monotherapy. Much work remains to be done to define optimal regimens and determine effects related to the full spectrum of ASCVD apart from CAD (Table **2**).

## 4 MECHANICAL HEART VALVES

Valvular heart disease has undergone many advances over the past decade, with the widespread use of transcatheter aortic valve interventions (TAVI) and the expansion of transcatheter techniques for mitral and tricuspid valve disease. While robust data are lacking for antithrombotic regimens in bioprosthetic valves, the 2020 ACC/AHA Guideline for the Management of Patients with Valvular Heart Disease recommendations for mechanical valves are clear - lifelong anticoagulation with warfarin - with proven, Class 1/A evidence of protection against valve thrombosis (OR 0.11; 95% CI 0.07 - 0.20) and thromboembolic events (OR 0.21; 95% 0.16 - 0.27) [7]. A major update in this 2020 statement revises the prior 2014 Class 1/A recommendation to use low-dose aspirin in addition to warfarin in mechanical valves to Class 2b based on a 2013 Cochrane Systematic Review finding of low-quality evidence from early trials starting in 1971 that shows a reduction in thromboembolic events and total mortality, but at an offsetting cost of significant increases in major bleeding (OR 1.58, 95% 1.14 - 2.18, p = 0.006) warranting careful consideration of bleeding risks in patients with mechanical valves and concomitant reasons for antiplatelet therapy [6]. The European Society of Cardiology/European Association of Cardiothoracic Surgery (ESC/EACTS) 2021 guidelines for managing valvular heart disease also reflect this update, reserving the combination of warfarin and an antiplatelet agent only for individuals at very high risk of thromboembolism [83]. The only exception to this recommendation surrounds the newer, mechanical On-X aortic heart valve (On-X Life Technologies, Austin, TX) with positive RCT data comparing a lower-intensity warfarin INR (1.5 - 2.0) goal to standard-intensity (2.0 - 3.0) both in combination with daily aspirin 81 mg to significantly reduce major and minor bleeding while maintaining similar efficacy in preventing major adverse cardiovascular events. In a separate On-X study, DAPT was compared to the low-intensity warfarin INR and aspirin 81 mg combination and found to cause harm, emphasizing the need for a careful balance of antithrombotic regimens among even the newest generation mechanical valves. Several different strategies have been evaluated following surgical or percutaneous bioprosthetic valve implantation, with much still to be learned about the risks and benefits of each. Recent studies comparing DAPT *vs.* low-dose aspirin appear to have comparable rates of thrombotic events with less bleeding in the low-dose aspirin monotherapy group [84-86]. Alternative regimens, including an initial 3 - 6 months of warfarin use among the Partner 2 TAVI investigation, have shown efficacy, while the Galileo study was stopped early due to harm induced with a low-dose rivaroxaban and aspirin combination compared to DAPT [87, 88]. Unlike mechanical valves, low-dose aspirin seems to have a solidified role in antithrombotic therapy for bioprosthetic valves.

## 5 PREECLAMPSIA

**Fig. (1):**
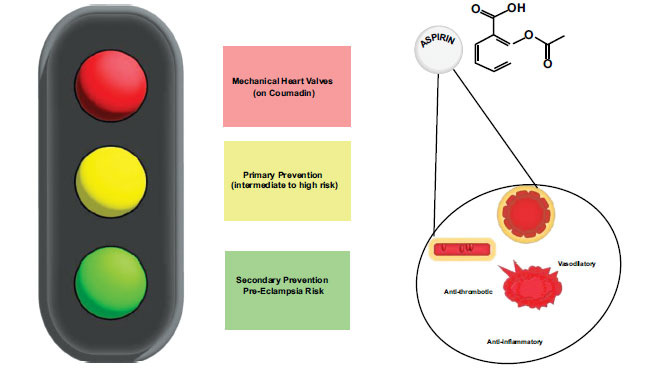
Aspirin in the current guidelines for the prevention of atherosclerotic cardiovascular disease.

Recently, the USPSTF further strengthened its original 2014 recommendation for the use of aspirin 81 mg daily after 12 weeks gestation for women at high risk for preeclampsia by including evidence of risk reduction not only for preeclampsia, but also for preventing preterm birth, small for gestational age/intrauterine growth restriction (SGA/IUGR), and perinatal mortality [8]. Clear high-risk criteria for preeclampsia were identified in the recommendation statement, and moderate risk factors were updated to consider socioeconomic and racial inequities. A total of 16 RCTs (n = 15,767) evaluated preeclampsia prevention, 18 RCTs (n = 15,908) evaluated maternal and perinatal health outcomes, and 21 RCTs (n = 26,757) evaluated potential maternal, perinatal, and developmental harms of daily aspirin use during pregnancy were considered [89-108]. Aspirin initiation throughout the RCTs ranged from 11 to 32 weeks - most before 20 weeks - at doses from 50 to 150 mg daily. With the additional trials since the largest performed in 1994 - Collaborative Low-Dose Aspirin Study in Pregnancy (CLASP) - the USPSTF found pooled relative risk reductions for preterm birth (13 trials, n = 13 619, 0.80 [95% CI 0.67 - 0.^95^]), SGA/IUGR (16 trials, n = 14 385, 0.82 [95% CI 0.68 - 0.^99^]), perinatal mortality (11 trials, n = 13 860, 0.79 [95% CI 0.66 - 0.^96^]), and preeclampsia (16 trials, n = 14 093, 0.85 [95% CI 0.75 - 0.^95^]) without any significant evidence of harm including placental abruption, postpartum hemorrhage, fetal intracranial bleeds, or physical/developmental abnormalities [8, 96, 98, 106-108]. This recommendation is also endorsed by the World Health Organization, AHA/American Stroke Association, and both the American College of Obstetricians and Gynecologists and the Society for Maternal-Fetal Medicine, who suggest aspirin initiation between 12 to 28 weeks gestation, ideally before 16 weeks, with continuation until delivery (Fig. **1**).

## CONCLUSION AND SUMMARY POINTS

The introduction of aspirin into cardiovascular care during the late 1970s and early 1980s was a major advance in treating ACS and secondary prevention of ASCVD. With robust results and ready availability, aspirin was rapidly incorporated into the treatment algorithm for various cardiovascular conditions. Aspirin became the standard for comparison among the Antiplatelet Trialists’ Collaboration with the 1994 publication providing insight into a smaller impact among 30,000 “low-risk” patients and the 2002 review shedding light on clopidogrel as an effective alternative [37, 38]. Over the years, other advances in cardiovascular medicine, such as statin therapy, understanding of the importance of neurohormonal blockade in cardiovascular remodeling, and continuous improvements in drug-eluting stent technology and revascularization techniques further shaped primary and secondary ASCVD prevention strategies to begin to redefine the role of aspirin. As ARRIVE, ASCEND, and ASPREE showed in the series of recent primary prevention studies, aspirin has the potential for more harm than good when not used in the right patient population. Importantly, guidelines and recommendations have been adapted to identify groups that benefit most. The importance of emphasizing the difference between primary and secondary prevention indications cannot be understated, as significant confusion ensued with recent updates in primary prevention recommendations. Aspirin has a steadfast role in the short-term treatment and prevention of recurrence of ASCVD, though its role in long-term secondary prevention ≥ 1 year may change with excess bleeding compared to P2Y12 inhibitors, as well as when used in combination with anticoagulants for other indications such as atrial fibrillation. This heightened bleeding risk combined with anticoagulation, particularly warfarin, is why aspirin’s importance was downgraded in the latest mechanical heart valve antithrombotic regimen guideline statement. Over the years, aspirin has proven to be the greatest net clinical benefit among those with the highest risk for cardiovascular disease or the highest burden of cardiovascular disease. With the advancement of revascularization technology, emphasis on smoking cessation and healthy lifestyles, and incorporation of alternative guideline-directed medical therapy such as statins, beta-blockers, and sodium-glucose co-transporter 2 (SGLT2) inhibitors, those at highest cardiac risk can be detected early and intervened upon sooner contributing to the diminishing role of aspirin in primary and secondary prevention of ASCVD. Aspirin remains a cornerstone of the cardiovascular realm but with emerging risk/benefit ratio data. Aspirin has found a new role in preventing preeclampsia, preterm birth, SGA/IUGR, and overall perinatal mortality in high-risk women.

## Figures and Tables

**Table 1:** Summary of largest aspirin for primary prevention of atherosclerotic cardiovascular disease randomized controlled trials.

**Trial (Year)**	**AsA dose**	**Population**	**Duration**	**Patients**	**RR/HR (95% CI), AsA/Placebo Events** **CVD Mortality ----------------------- ASCVD Affects ------------------- Risks**		
PHS (1989)	325 mg qOd	Men	5 years	22,071	0.96 (0.60-1.54), 81/83	Myocardial Infarction 0.56 (0.45-0.70), 255/440	Blood transfusion need 1.71 (1.09-2.69), 48/28 Hemorrhagic Stroke 2.14 (0.96-4.77), 23/12 Upper GI Ulcers 1.22 (0.98-1.53), 169/138
WHS (2005)	100 mg qOd	Women	10 years	39,876	0.95 (0.74-1.22), 120/126	Stroke *(ischemic driven)* 0.83 (0.69-0.99), 221/266 MACE 0.91 (0.80-1.03), 477/522	GIB requiring transfusion 1.40 (1.07-1.82), 127/91 Hemorrhagic Stroke 1.24 (0.82-1.87), 51/41
ASCEND (2018)	100 mg qD	Diabetic	7.4 years	15,480	0.91 (0.75-1.10), 197/217	Fatal/Non-Fatal ASCVD 0.88 (0.79-0.97), 658/743	Major Bleeding 1.29 (1.09-1.59), 314/245
ASPREE (2018)	100 mg qD	Elderly	4.7 years	19,114	0.97 (0.71-1.33), 78/81	Disability-Free Survival 0.95 (0.83-1.08), 10.7/11.3	Major Hemorrhage 1.38 (1.18-1.62), 8.6/6.2 Death From Any Cause 1.14 (1.01-1.29), 12.7/11.1
ARRIVE (2018)	100 mg qD	Mod Risk	5 years	12,456	0.97 (0.62-1.52), 38/39	Fatal/Non-Fatal ASCVD 0.96 (0.81-1.31), 269/281	GIB (mostly mild) 2.11 (1.36-3.28), 61/29

Note: *Bolding indicates statistically significant variables. **Abbreviations:** GI = gastrointestinal; GIB = gastrointestinal bleeding; qD = every day; qOd = every other day; RR = relative-risk reduction; HR = hazard ratio.

**Table 2:** Review of Important Meta-Analyses on Aspirin Use for Secondary Prevention of Atherosclerotic Cardiovascular Disease.

**Review** **(PUB, Year)**	**Objective**	**Studies**	**Patients**	**Agents** **(# patients, %)**	**Key Findings, OR ± SD; HR/OR/RR (95% CI)** **Data --------------------------------------- Conclusions**	
ATT part I (BMJ, 1994)	AP Role in ASCVD	145 RCTs: AP *vs.* Placebo (C) [+] 29 RCTs: ASA *vs* AP(s)	Total: 102,459 High Risk: 73,247 - 36,536 AP - 36,711 C Low Risk: 29,212	ASA mg (22,471) 500-1500: 27% 160-325: 33% <160: 4% Others (13,689) ASA + Dipyr:18% Ticlodipine: 9% Sulphinpyr: 6% Dipyr: 2% Suloctidil: 1% ASA + Sulph: 1%	Death from any cause (high-risk group) AP 2929 (8.0%) *vs.* C 3465 (9.5%), OR 17% ± 3%, p < 0.01 Non-Fatal MI/Stroke or Vascular Death All: AP 4835 (9.5%) *vs* C 6108 (11.9%), OR 25% ± 2%, p < 0.0001 AMI: 10.6% *vs* 14.4% (OR 29% ± 4%) H/o MI: 13.5% *vs* 17.1% (OR 25% ± 4% H/o CVA: 18.4% *vs* 22.2% (OR 22% ± 4%) Low: 4.5% *vs* 4.8% (OR 10% ± 6%), p = .09 Vascular Events Across ASA Doses Any: 11.9% *vs* 16.2% (OR 25% ± 2%) 500-1500: 13.5% *vs* 16.5% (OR 21% ± 4%) 160-325: 11.0% *vs* 14.7% (OR 28% ± 3%) <160: 9.0% *vs* 11.7% (OR 16% ± 11%)	AP Therapy has definitive protective effects against recurrent vascular events Medium dose ASA (75-325 mg) is the most widely tested, and no other dose/AP regimen is significantly better Uncertain benefits among the low-risk population with a less absolute risk
ATT Update (BMJ, 2002)	AP Role in ASCVD	197 RCTs: AP *vs.* Placebo (C) [+] 90 RCTs: ASA *vs* AP(s)	Total: 216,064 High Risk: 135,640 AP Comp: 80,424	ASA mg (29,652) 500-1500 38% 160-325 45% 75-150 11% <75 6% ASA *vs* ASA ~6K >500 *vs* 75-325 ≥ 75 *vs* < 75 vs ASA (36,711) IV GP+ASA 37% Clopidogrel 26% Dipyr+ASA 14%	Vascular Events Across AP Agents 14.5% *vs* C 17.2% (OR 19% ± 3%) 11.5% *vs* C 14.8% (OR 26% ± 3%) 10.9% *vs* C 15.2% (OR 32% ± 6%) 17.3% *vs* C 19.4% (OR 13% ± 8%) 14.1% *vs* 14.5% (OR 3% ± 10%), ns 14.2% *vs* 13.2% (OR 8 ± 10%), ns 9.9% *vs* 11.8% (OR 19% ± 4%) 10.1% *vs* 11.1% (OR 10% ± 4%) 11.8% *vs* 12.4% (OR 6% ± 6%), ns	ASA 75-150 mg doses appear as effective as higher doses Transient Intravenous glycoprotein IIb/IIIa inhibitor addition to ASA reduces vascular events at the risk of extracranial bleeding Clopidogrel is a suitable alternative to ASA
O’Donoghue *et al.* (Circ, 2020)	DAPT Duration 1-3 months DAPT after PCI before stopping ASA	5 RCTs GLOBAL LEADERS SMART CHOICE STOPDAPT2 TWILIGHT TICO	Total: 32,145 56.1% ACS 43.8% CCD Follow-up: 12-15 mo	Clopidogrel 2,649 (16.5%) Prasugrel/ Ticagrelor 13,408 (83.5%) +/- ASA 75-100 mg	P2Y12i Monotherapy 1-3 mo *vs.* DAPT Primary Bleeding Outcome 0.60 (0.45 - 0.79), 317/503 Major Bleeding (BARC 3/5) 0.60 (0.42 - 0.86), 196/291 Primary MACE Outcome 0.88 (0.77 - 1.02), 438/499 Stent Thrombosis 1.17 (0.84 - 1.63), 80/67	Reassurance that ASA can be safely discontinued 1-3 months after PCI without an increase in MACE Reduced risk of bleeding with P2Y12i monotherapy after 1-3 months as opposed to traditional DAPT
Knijnik et Al (AJC, 2021)	DAPT Duration 1-3 mo DAPT after PCI before stopping ASA in purely ACS	5 RCTs GLOBAL LEADERS SMART CHOICE STOPDAPT2 TWILIGHT TICO	Total: 18,046 100% ACS 22% STEMI Follow-up: 12 months	Clopidogrel 2,052 (11%) Ticagrelor 15,994 (89%) +/- ASA 75-100 mg	P2Y12i Monotherapy 1-3 mo *vs.* DAPT Major Bleeding 0.53 (0.42 - 0.67), 113/213 Primary MACE Outcome 0.86 (0.71 - 1.03), 223/262 Net Clinical Outcome (MACE + Bleeding) 0.81 (0.72 - 0.91), 476/580 All-cause Mortality 0.71 (0.53 - 0.95), 76/108	Among patients with PCI for ACS, shorter DAPT of 1-3 months before ASA discontinuation and P2Y12i monotherapy reduces bleeding and does not worsen MACE A mortality benefit was seen among this ACS shorter-DAPT population
Aggarwal *et al.* (EHJ, 2022)	Maintenance AP Agent after ANY ASCVD	9 RCTs: ASCVD CAPRIE: CVA, CAD, PAD GLOBAL LEADERS: CAD s/p PCI SOCRATES: CVA/TIA HOST-EXAM: CAD s/p PCI CHANCE: CVA/TIA TICAB: CAD s/p CABG ASCET: CCD DACAB: CAD s/p CABG CADET: AMI	Total: 61,623 19,185 15,968 13,199 5,438 5,170 1,859 1,001 332 184	P2Y12i (30,844) Clopidogrel 5 studies Ticagrelor 4 studies ASA (30,779) 75 - 325 mg daily Monotherapy Duration: 68 days - 36 mo Follow-up Duration: 3 mo - 36 mo	P2Y12i Monotherapy *vs.* ASA Primary MACE Outcome 0.89 (0.84 - 0.95), 1,827/2,044 Myocardial Infarction 0.81 (0.71 - 0.92), 427/531 Stroke 0.85 (0.73 - 1.01), 916/1,033 All-Cause Mortality (0.92 - 1.11), 827/820 Major Bleeding 0.94 (0.72 - 1.22), 283/310	Across various ASCVD events, P2Y12i monotherapy significantly reduced MACE and rates of MI compared with ASA monotherapy No difference in rates of stroke, all-cause mortality, or major bleeding between either monotherapy
PANTHER (ESC, 2022)	Maintenance AP Agent specifically after CAD	7 RCTs CAPRIE GLASSY HOST-EXAM TICAB ASCET DACAB CADET	Total: 24,325 8,446 7,065 5,438 1,859 1,001 332 184	P2Y12i (12,178) Clopidogrel (7,545; 62%) Ticagrelor (4,633; 38%) ASA (12,147) 75 - 325 mg daily Follow-up Duration: ~ 18 months	P2Y12i Monotherapy *vs.* ASA Primary Efficacy Outcome: MACE 0.88 (0.79 - 0.97), 5.5% *vs.* 6.3% Primary Safety Outcome: Major Bleeding 0.87 (0.70 - 1.09), 1.2% *vs.* 1.4% Key Secondary Outcome: Net Adverse Clinical Events 0.89 (0.81 - 0.98), 6.4% *vs.* 7.2% Notable Secondary Outcomes: Myocardial Infarction 0.77 (0.66 - 0.90), p < 0.001 Definite Stent-Thrombosis 0.42 (0.19 - 0.97), p = 0.041 Definite/Probable Stent-Thrombosis 0.46 (0.23 - 0.92), p = 0.028 Hemorrhagic Stroke 0.32 (0.14 - 0.75), p = 0.009 Gastrointestinal Bleeding 0.75 (0.57 - 0.97), p = 0.027	P2Y12i monotherapy was associated with lower risks of MACE in patients with CAD compared to ASA monotherapy Significant reductions in MI, definite/probable stent-thrombosis, hemorrhagic stroke, and GIB with P2Y12i monotherapy contributed to a reduction in NACE For secondary prevention in patients with established CAD, data suggests significant benefits of P2Y12i monotherapy over ASA for long-term maintenance therapy

*Bolding indicates statistically significant variables. Abbreviations: ATT = antithrombotic trialists’ collaboration; PUB = publication/journal; OR = odds ratio; SD = standard deviation; HR = hazard ratio; RR = relative risk; RCTs = randomized-control trials; AP = antiplatelet; C = control/placebo; ASA = aspirin; AMI = acute myocardial infarction; H/o = history of; MI = myocardial infarction; CVA = cerebrovascular accident/stroke; Dipyr = dipyridamole; Sulphinpyr/Sulph = sulphinpyrazone; IV GP = intravenous glycoprotein IIb/IIIa antagonist; MACE = major adverse cardiovascular event; P2Y12i = P2Y12 inhibitor; BARC = Bleeding Academic Research Consortium; DAPT = dual-antiplatelet therapy; CAD = coronary artery disease; CABG = coronary artery bypass grafting; PCI = percutaneous coronary intervention; PAD = peripheral artery disease; mo = month(s); NACE = net adverse clinical events; GIB = gastrointestinal bleeding.

## LIST OF ABBREVIATIONS

AP: Antiplatelet
ASCVD: Atherosclerotic cardiovascular disease
CAD: Coronary artery disease
CVA: Cerebrovascular accident
HR: Hazard ratio
MI: Myocardial infarction
PAD: Peripheral arterial disease
RR: Relative risk
TIA: Transient ischemic attack
USPSTF: the United States Preventive Services Task Force
